# Early pannus formation

**DOI:** 10.11604/pamj.2023.44.187.39601

**Published:** 2023-04-20

**Authors:** Ismail Oughebbi

**Affiliations:** 1 Department of Cardiovascular Surgery, Ghassani Hospital, Fes, Morocco

**Keywords:** Early, pannus, valve, deterioration

## Image in medicine

Structural valve deterioration is commonly defined as an intrinsic permanent change of the bioprosthesis due to leaflet calcification, thickening, pannus formation, tear, or disruption. The resulting deterioration leads to stenosis and/or intra-prosthetic regurgitation. Here we present the case of a 75-year-old patient who underwent aortic valve replacement with a bioprosthetic valve. Predischarge transthoracic echocardiography revealed an aortic prosthesis with normal gradients. Three years later, the patient reported exertional dyspnea. Transthoracic echocardiography was performed and showed a high transvalvular pressure gradient of 80 mmhg with restricted mobility of the leaflet caused by subprosthetic tissue. Redo aortic valve replacement was planned and surgical resection was performed showing pannus ingrowth in both aortic and ventricular side of the bioprosthesis.

**Figure 1:**
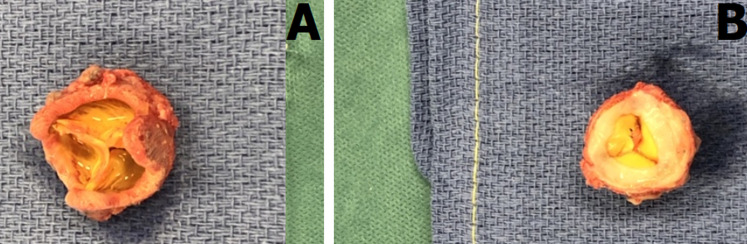
surgical specimen of the bioprosthetic aortic valve; A) circumferential fibrous tissue ingrowth in both aortic; B) ventricular side compatible with pannus

